# Risk factors for surgical site infection after craniotomy: a prospective cohort study

**DOI:** 10.1186/s13756-019-0525-3

**Published:** 2019-05-02

**Authors:** Emilio Jiménez-Martínez, Guillermo Cuervo, Ana Hornero, Pilar Ciercoles, Andres Gabarrós, Carmen Cabellos, Ivan Pelegrin, Dolores García-Somoza, Jordi Adamuz, Jordi Carratalà, Miquel Pujol

**Affiliations:** 1 Infectious Diseases Department, Institut d’Investigació Biomèdica de Bellvitge (IDIBELL), Bellvitge University Hospital, Feixa Llarga s/n, 08907, L’Hospitalet de Llobregat, Barcelona, Spain; 2 Neurosurgery Department, Bellvitge University Hospital-Institut d’Investigació Biomèdica de Bellvitge (IDIBELL), Barcelona, Spain; 3 Infectious Diseases Department, H. Parc Taulí, Sabadell, Spain; 4 Microbiology Department, Bellvitge University Hospital-Institut d’Investigació Biomèdica de Bellvitge (IDIBELL), Barcelona, Spain; 5 Nursing Information Systems Department Support, Bellvitge University Hospital-Institut d’Investigació Biomèdica de Bellvitge (IDIBELL), Barcelona, Spain; 6 University of Barcelona, Barcelona, Spain

**Keywords:** Risk factors, Surgical site infection, Craniotomy

## Abstract

**Background:**

Although surgical site infection after craniotomy (SSI-CRAN) is a serious complication, risk factors for its development have not been well defined. We aim to identify the risk factors for developing SSI-CRAN in a large prospective cohort of adult patients undergoing craniotomy.

**Methods:**

A series of consecutive patients who underwent craniotomy at a university hospital from January 2013 to December 2015 were prospectively assessed. Demographic, epidemiological, surgical, clinical and microbiological data were collected. Patients were followed up in an active post-discharge surveillance programm e for up to one year after surgery. Multivariate analysis was carried out to identify independent risk factors for SSI-CRAN.

**Results:**

Among the 595 patients who underwent craniotomy, 91 (15.3%) episodes of SSI-CRAN were recorded, 67 (73.6%) of which were organ/space. Baseline demographic characteristics were similar among patients who developed SSI-CRAN and those who did not. The most frequent causative Gram-positive organisms were *Cutibacterium acnes* (23.1%) and *Staphylococcus epidermidis* (23.1%), whereas *Enterobacter cloacae* (12.1%) was the most commonly isolated Gram-negative agent. In the univariate analysis the factors associated with SSI-CRAN were ASA score > 2 (48.4% vs. 35.5% in SSI-CRAN and no SSI-CRAN respectively, *p* = 0.025), extrinsic tumour (28.6% vs. 19.2%, *p* = 0.05), and re-intervention (4.4% vs. 1.4%, *p* = < 0.001). In the multivariate analysis, ASA score > 2 (AOR: 2.26, 95% CI: 1.32–3.87; *p* = .003) and re-intervention (OR: 8.93, 95% CI: 5.33–14.96; *p* < 0.001) were the only factors independently associated with SSI-CRAN.

**Conclusion:**

The risk factors and causative agents of SSI-CRAN identified in this study should be considered in the design of preventive strategies aimed to reduce the incidence of this serious complication.

**Electronic supplementary material:**

The online version of this article (10.1186/s13756-019-0525-3) contains supplementary material, which is available to authorized users.

## Background

Craniotomy is a surgical procedure in which part of the skull bone is removed to expose the brain and the central nervous system. The bone flap is temporarily separated and is returned to its previous location at the end of surgery in order to protect the brain and its structures. In many cases, metal plates are used to hold the bone flap in place. The incidence of surgical site infection after craniotomy (SSI-CRAN) ranges from 2.2 to 19.8% [1–6]. SSI-CRAN has potentially devastating consequences: it is associated with significant morbidity and requires complex treatment that often involves the removal of the bone flap and long-term antibiotic therapy [5, 7]. Indeed, SSI-CRAN is associated with longer hospital stay, significant health care costs, and non-negligible mortality [1].

The risk factors for SSI-CRAN are still poorly understood. Previous studies have identified several associated factors, including age, gender, duration of operation, surgical site, reason for surgery, emergency procedure, antibiotic prophylaxis, steroid use, cerebrospinal fluid (CSF) drainage, and American Society of Anesthesiologists (ASA) score [2–12]. However, those studies presented certain limitations, such as small sample sizes, substantial variations in the inclusion criteria, and marked differences in patients’ baseline characteristics. Therefore, more evidence is still needed to define more precisely the risk factors for developing SSI-CRAN. An accurate identification of the risk factors and main causative pathogens of SSI-CRAN might be helpful in the design of future preventive strategies.

In this study, we aimed to identify the aetiology and risk factors of SSI-CRAN in a large contemporary cohort of consecutive adult patients undergoing craniotomy in a university hospital.

## Methods

### Study design, setting and patients

A prospective cohort study was carried out in a 700-bed university hospital for adults in Barcelona, Spain, which admits average of 1300 patients to the neurosurgery ward each year. All adult patients (> 18 years old) who underwent a craniotomy (e.g., tumour resection, epilepsy, vascular abnormalities, trauma, etc.) from 1st January 2013 to 31th December 2015 were included. Patients were prospectively followed up by members of the hospital infection control team. The staff performing surveillance had received training in surveillance methodology to ensure the collection of homogeneous and accurate data. Active mandatory post-discharge surveillance was carried out up to 1-year post surgery applying a multimodal approach including the following items: a) electronic review of clinical records (primary and secondary care) integrated in the platform SAP® (Systems, Applications & Products, Waldorff, Germany); b) checking of readmissions; c) checking of emergency visits; d) review of microbiological and radiological data within the period of post-discharge surveillance.

All patients underwent the same protocol regarding preparation for surgery, which include at least showering (with 4% clorhexidine gluconate detergent solution) on the day before and on the morning of the operation, head included. In the surgical area, the hair was cut with a sterile electric clipper. Pre-operative skin preparation involved standardized application of at least three swabs, soaked with povidone-iodine solution. Antibiotic prophylaxis involved pre-operative intravenous cefuroxime 1500 mg 30–60 min prior to incision and re-administration every 3 h during the operation. The skin was closed using a skin stapler and the head was washed with povidone-iodine solution. Post-operatively, the surgical wound was draped with a sterile impermeable towel for the first 24 h post-surgery. Afterwards, a head wash was performed every 12 h with povidone-iodine soap the first 72 h and surgical wound-care was carried out every 24 h under strict aseptic conditions. Povidine-iodine was used instead of 2% clorhexidine because of neurotoxicity [13].

At the moment of SSIs diagnosis, neurosurgeons and infectious diseases specialists work side by side in the management of these patients. In superficial incisional SSIs, treatment often involves specific wound care with silver-containing hydrofiber dressings and sometimes additional systemic antibiotic treatment. In deep incisional and organ-space SSIs, surgical re-intervention is mostly needed, involving bone graft replacement and an invariably systemic antibiotic treatment.

The need for informed consent and the information sheet were waived because the follow-up of patients undergoing craniotomy is part of the centre’s own surveillance programme. Ethical standards related to anonymity and data confidentiality (access to records, data encryption, and archiving of information) were observed throughout the research process. Patients’ confidential information was protected in accordance with European regulations, and the study was approved by the Clinical Research Ethics Committee of Bellvitge University Hospital (Reference number PR334/18).

### Main outcome, variables, and data source

The main outcome analysed was the development of a SSI-CRAN within one year post-surgery. The clinical characteristics of patients who developed SSI-CRAN were compared with those who did not. Basic demographic data were recorded, along with the following information on patient comorbidities and surgical procedure: Charlson score, diabetes mellitus, chronic obstructive pulmonary disease, immunosuppression and cirrhosis; information on surgical procedures, including ASA and National Nosocomial Infections Surveillance (NNIS) score, elective/emergency surgery, reason for surgery (intrinsic tumour, extrinsic tumour, epilepsy, vascular, traumatic or others), operative site (supratentorial, infratentorial or retromastoid), administration of antibiotic prophylaxis according to hospital guidelines, duration of surgery, use of intracranial pressure (ICP) monitors, number of surgeries, CSF leak and metal plates; characteristics of infection (SSI-CRAN classification and microorganisms identified); and in-hospital outcome data (pre and post-surgery in-hospital stay).

### Definitions

SSI-CRAN was defined according to Centers for Disease Control and Prevention (CDC) criteria, as follows: a) purulent drainage from a surgical incision; b) organism identification by culture from fluid or tissue obtained aseptically; c) incision that dehisces spontaneously or is deliberately opened by a surgeon or physician, localized pain or tenderness, localized inflammation (heat, erythema and swelling), and/or fever (> 38 °C); and evidence of abscess on images or surgical revision. SSI-CRAN was also classified according to CDC criteria as superficial incisional, deep incisional or organ-space infection [14]. Post-discharge surveillance of SSI was mandatory and consisted in the review of electronic clinical records in primary and secondary care, checking readmissions and emergency visits, and reviewing microbiological and radiological data.

The reason for surgery was defined by the patient’s disease, which was divided into: a) intrinsic tumour in the parenchyma, b) extrinsic tumour in the structures of the central nervous system in the skull, c) epilepsy, d) vascular disease, e) traumatic disease and f) others. The use of steroids/chemotherapy both in the last two weeks pre-surgery and in the two weeks post-surgery was recorded.

Charlson Score was defined as a system of evaluation of life expectancy at ten years, depending on the age at which it is evaluated, and the comorbidities of the subject [15]. Intravenous antibiotic prophylaxis was considered adequate when the following three factors were all met: antibiotic administration according to local protocol, completion of the infusion within 60 min of the surgical incision, and perioperative antibiotic re-administered if indicated.

### Microbiological studies

In most patients with suspected SSI-CRAN, microbiological samples from wounds and/or CSF fluid or abscesses were taken for culture. Blood cultures were also taken when indicated by the attending physician. Antibiotic susceptibility was determined using the microdilution method following Clinical Laboratory Standard Institute (CLSI) guidelines. The antimicrobial susceptibility of isolates was interpreted according to current CLSI criteria [16].

### Statistical methods

Quantitative variables are reported as medians and interquartile range (IQR); categorical variables are reported as absolute numbers and percentages. To detect significant differences between groups, the chi-square test or the Fisher’s exact test was used for categorical variables, and the Student *t-*test or Mann–Whitney *U* test for continuous variables, as appropriate. Factors associated with SSI-CRAN were evaluated by univariate and multivariate analysis. The multivariate analysis included variables from the univariate analysis with a *p*-value < 0.1 and other factors considered relevant according to a literature review. The goodness of fit of the final multivariate model was assessed by the Hosmer–Lemeshow test and the area under the receiver operating characteristics curve. Results of multivariate analyses were reported as odds ratios (OR) and 95% confidence intervals (CI). The statistical analysis was performed with version 24.0 of the SPSS software package (SPSS, Chicago, IL). Statistical significance was established at α = 0.05, and all reported *p*-values are two-tailed.

This paper was written in accordance with the Additional file 1: STROBE statement (https://strobe-statement.org/index.php?id=available-checklists).

## Results

During the study period, 595 patients who underwent craniotomy were followed up. Baseline clinical characteristics are summarized in Table 1. There were 274 male patients (46.1%) and mean age was 52.8 years (standard deviation [SD] = 14.4). An average of 198 interventions were performed each year during the study period. The main causes for surgical intervention were intrinsic tumour (*n* = 234, 39.3%), and vascular disease (*n* = 152, 25.5%). Supratentorial region was the most common frequent surgical site (*n* = 449, 75.5%). Of the 91 episodes of SSI-CRAN, 61 episodes (67%) required re-intervention.

**Table 1:** Baseline characteristics

Baseline characteristics		
	OVERALL (*n* = 595)	
Male, *n* (%)	274	(46.1)
Mean age, years (SD)	52.8	(14.4)
*> 65y, n (%)*	148	(24.9)
Urgent surgery, *n* (%)	79	(13.3)
Reason of surgery:		
*Intrinsic tumour*, *n* (%)	234	(39.3)
*Extrinsic tumour*, *n* (%)	123	(20.7)
*Epilepsy*, *n* (%)	23	(3.9)
*Vascular*, *n* (%)	152	(25.5)
*Trauma*, *n* (%)	17	(2.9)
*Others*, *n* (%)	46	(7.7)
Surgical site:		
*Supratentorial*, *n* (%)	449	(75.5)
*Infratentorial*, *n* (%)	62	(10.4)
*Retromastoid*, *n* (%)	84	(14.1)
Duration of surgery, minutes (SD)	256.9	(101.5)
*> P75*^a^, *n* (%)	152	(25.5)
Mean hospital stay, median (range)	7	(5–14)
CHARLSON score, (range)	3	(0–13)
*< 3*, *n* (%)	292	(49.1)
*3–5*, *n* (%)	214	(36)
*6–8*, *n* (%)	69	(11.6)
*> 9*, *n* (%)	20	(3.4)
ASA^b^, (range)	2	(1–5)
*ASA > 2, n (%)*	223	(37.5)
NNIS^c^, (range)	1	(0–2)
*NNIS I-II, n (%)*	441	(74.1)

^a^ > 75 percentile of duration of surgery

^b^American Society of Anaesthesiologists

^c^National Nosocomial Infection Surveillance System

The overall SSI-CRAN incidence at the end of follow-up was 15.3% (*n* = 91). As shown in Table 2, SSI was organ-space in 73.6% (*n* = 67) of patients, deep-incisional in 17.6% (*n* = 16) and superficial in 8.8% (*n* = 8). Most of the SSI-CRAN required rehospitalization (*n* = 49, 53.8%). The aetiology varied according to surgical site: overall Cutibacterium acnes (*n* = 21, 23.1%) and *Staphylococcus epidermidis* (n = 21, 23.1%) were the most frequently isolated Gram-positive microorganisms, whereas *Enterobacter cloacae* (*n* = 11, 12.1%) was the most prevalent Gram-negative microorganism (Table 3). According to surgical site, C. acnes was the most frequent aetiology in retromastoid and supratentorial areas (33.3 and 23.8% respectively), while methicillin-susceptible *Staphylococcus aureus* was the most commonly isolated organism in infratentorial regions (38.5%). No differences were found between aetiology and reason of surgery.

**Table 2:** Characteristics of SSI-CRAN

SSI-CRAN *(n = 91)*		
Occurrence of SSI-CRAN, days (SD)	39	(54)
Detection:		
*During hospital admission*, *n* (%)	31	(34.1)
*Post-discharge surveillance*, *n* (%)	11	(12.1)
*Readmission*, *n* (%)	49	(53.8)
SSI-CRAN classification:		
*Superficial*, *n* (%)	8	(8.8)
*Deep incisional, n* (%)	16	(17.6)
*Organ-space*, *n* (%)	67	(73.6)
Age, mean (SD)	51.4	(15.3)

**Table 3:** SSI-CRAN aetiology

SSI-CRAN aetiology									
		Supratentorial (*n* = 63)		Infratentorial (*n* = 13)		Retromastoid (*n* = 15)		Overall (*n* = 91)	
		*n*	%	*n*	%	*n*	%	*n*	%
GPC^a^	*Cutibacterium acnes*	15	(23.8)	1	(7.7)	5	(33.3)	21	(23.1)
	*Staphylococcus epidermidis*	13	(20.7)	4	(30.8)	4	(26.7)	21	(23.1)
	*MSSA* ^c^	11	(17.5)	5	(38.5)	2	(13.3)	18	(19.8)
	*MRSA* ^d^	3	(4.8)	0	–	0	–	3	(3.3)
	*CNS* ^e^	1	(1.6)	0	–	2	(13.3)	3	(3.3)
GNB^b^	*E.cloacae*	9	(14.3)	1	(7.7)	1	(6.7)	11	(12.1)
	*P.aeruginosa*	5	(8)	2	(15.4)	1	(6.7)	8	(8.8)
	*K.pneumoniae*	4	(6.3)	1	(7.7)	0	–	5	(5.5)
	*E.coli*	3	(4.8)	1	(7.7)	0	–	4	(4.4)
Polymicro (>2microorg)		1	(1.6)	0	–	1	(6.7)	2	(2.2)
Others		17	(27)	3	(23.1)	4	(26.7)	24	(26.4)

^a^Gram-positive cocci

^b^Gram-negative bacilli

^c^methicillin-susceptible *Staphylococcus aureus*

^d^methicillin-resistant *Staphylococcus aureus*

^e^Coagulase Negative Staphylococci

A comparison of the study population by groups (SSI-CRAN and non-SSI-CRAN) is shown in Table 4.

**Table 4:** Univariate and multivariate analysis of risk factors for treatment failure in organ-space SSI-CRAN

Univariate and multivariate analysis of risk factors for developing SSI-CRAN								
	UNIVARIATE					MULTIVARIATE		
	Non SSI-CRAN (*n* = 504)		SSI-CRAN (*n* = 91)		*p*-value	OR	95% CI	p-value
Male, *n* (%)	232	(46)	42	(46.2)	1	0.86	0.52–1.43	0.57
Mean age, years (SD)	53.1	(14.3)	51.4	(15.3)	0.26	0.99	0.97–1.01	0.33
*> 65 years (%)*	129	(25.6)	19	(20.9)	0.36			
Urgent surgery, *n* (%)	66	(13.1)	13	(14.3)	0.74			
Reason for surgery:								
*Intrinsic tumour*, *n* (%)	203	(40.3)	31	(34.1)	0.29			
*Extrinsic tumour*, *n* (%)	97	(19.2)	26	(28.6)	**0.05**	1.71	0.91–3.24	0.09
*Epilepsy*, *n* (%)	23	(4.6)	0	(0)	**0.03**	0	0	0.99
*Vascular*, *n* (%)	130	(25.8)	22	(24.2)	0.79			
*Trauma*, *n* (%)	14	(2.8)	3	(3.3)	0.73			
*Others*, *n* (%)	37	(7.3)	9	(9.9)	0.39			
Surgical site:								
*Supratentorial*, *n* (%)	386	(76.6)	63	(69.2)	0.15			
*Infratentorial*, *n* (%)	49	(9.7)	13	(14.3)	0.19			
*Retromastoid*, *n* (%)	69	(13.7)	15	(27.5)	0.51			
Duration of surgery, minutes (SD)	255.2	(99.6)	265.9	(111.4)	0.65			
*> P75*^a^, *n* (%)	124	(24.6)	28	(30.8)	0.57			
Mean hospital stay, median (range)	7	(5–13)	7	(5–31)	0.59			
CHARLSON, mean (SD)	3.14	(2.3)	3.03	(2.2)	0.70	0.90	0.78–1.05	0.20
*< 3*, *n* (%)	245	(48.6)	47	(51.6)	0.65			
*3–5*, *n* (%)	184	(36.5)	30	(33)	0.55			
*6–8*, *n* (%)	56	(11.1)	13	(14.3)	0.38			
*> 9*, *n* (%)	19	(3.8)	1	(1.1)	0.34			
ASA^b^, mean (SD)	2.3	(0.7)	2.4	(0.8)	0.08			
*ASA > 2, n (%)*	179	(35.5)	44	(48.4)	**0.025**	2.26	1.32–3.87	**0.003**
NNIS^c^, mean (SD)	0.9	(0.7)	1	(0.7)	0.24			
*NNIS I-II, n (%)*	372	(73.8)	69	(75.8)	0.79			
Antibiotic prophylaxis:								
*Appropriate*, *n* (%)	432	(85.7)	80	(87.9)	0.74			
*Inappropriate*, *n* (%)	59	(11.7)	11	(12.1)	0.86			
*Non registered*, n (%)	13	(2.6)	0	(0)	0.23			
Metal plates, *n* (%)	465	(92.3)	78	(85.7)	0.06	1.8	0.84–3.87	0.13
Use of steroids/chemotherapy, *n* (%)	369	(73.2)	70	(76.9)	0.52			
Re-intervention, *n* (%)	97	(19.2)	61	(67)	**< 0.001**	8.93	5.33–14.96	**< 0.001**
ICP^d^ sensor, *n* (%)	30	(6)	6	(6.6)	0.81			
CSF^e^ leak, *n* (%)	7	(1.4)	4	(4.4)	0.72	0.85	0.21–3.44	0.82
Mortality 30d post surgery, *n* (%)	31	(6.2)	2	(2.2)	0.21			

^a^ > 75 percentile of duration of surgery

^b^American Society of Anaesthesiologists

^c^National Nosocomial Infection Surveillance System

^d^Intracranial pressure

^e^Cerebrospinal fluid

Univariate analysis of risk factors found higher ASA scores (ASA score > 2 [48.4% vs. 35.5%, *p* = 0.025]), extrinsic tumour (28.6% vs. 19.2%, *p* = 0.05) and re-intervention (4.4% vs. 1.4%, p = < 0.001) to be significantly associated with SSI-CRAN. Interestingly, metal plates was less frequent in SSI-CRAN group (92.3% vs. 85.7%, *p* = 0.06). Conversely, no difference were found between the groups in terms of emergency surgery (13.1% vs. 14.3%, *p* = 0.74), NNIS score I-II (73.8% vs. 75.8%, *p* = 0.79) and inappropriate antibiotic prophylaxis (11.7% vs. 12.1%, *p* = 0.86).

The multivariate logistic regression analysis (Hosmer–Lemeshow goodness of fit of the model: 0.713. AUC = 0.81; 95% CI, 0.77–0.86) showed that the only independent risk factors for developing a SSI-CRAN were ASA score > 2 (AOR: 2.26, 95% CI: 1.32–3.87; *p* = 0.003) and re-intervention (AOR: 8.93, 95% CI: 5.33–14.96; *p* < 0.001).

## Discussion

This study of a large cohort of hospitalized patients undergoing a craniotomy at a teaching hospital in Barcelona found that the most frequently isolated causative agents were Gram-positive cocci and that the only risk factors independently associated with SSI-CRAN were ASA score > 2 and need for re-intervention.

The overall SSI-CRAN rate was 15.3%. This percentage is higher than those reported in previous studies (a mean rate of 6.1%) [1, 6, 8, 12, 17]. In line with other studies, organ-space SSI-CRAN was the most prevalent surgical site infection [3, 4]. Despite the severity of the infections, mortality was low. On the other hand, there is a considerable increase in hospital days. The high rate of SSI-CRAN found in the current study could be partly explained by the use of different definitions and a stricter and longer patient follow-up than in previous research [18–21]. The CDC score is a well-established tool for the classification of surgical site infection and provides homogeneity among studies; however, it only includes surgical site infection within 30 days after surgery. In contrast, our data, with a follow up of one year, found the median time for the occurrence of SSI-CRAN to be 39 days (SD = 54); furthermore, most SSI-CRANs were detected in the post-discharge surveillance period, and frequently required hospital readmission. If the follow up was for 30 days the SSI-CRAN rate would be 58 episodes (9,7%), almost half of these episodes would have been lost. These findings concur with the results of other studies [5, 6] suggesting that limiting follow-up to 30-days would cause several cases to be missed. In our view, the CDC scoring system should be used, but with a minimum follow-up time of 3 months (See Additional file 2).

The most common reasons for surgery in our study were tumour (intrinsic and extrinsic) and vascular conditions. Rates of SSI-CRAN following extrinsic tumours surgery were significantly higher than following other surgeries: extrinsic tumours require a more aggressive approach and are often associated with difficulties in haemostasis and closure. Interestingly, there was no relation between SSI-CRAN and epilepsy. As most of the few studies on the subject [22–24] are case reports or have a small sample size, more evidence is still needed.

To the best of our knowledge, this is the first study evaluating SSI-CRAN aetiologies according to surgical site. The most frequently isolated microorganisms were Gram-positive cocci, as seen in many previous studies [2, 4, 5, 7, 8, 17]. C. acnes has frequently been identified as a cause of delayed SSI-CRAN [25]. These findings lend support to our proposal to extend post-discharge surveillance to a minimum of 3 months post-surgery, in order to avoid loss of cases. Interestingly, we found a higher rate of SSI-CRAN caused by *E. cloacae* than other studies [2, 4, 5, 7, 8, 17]. It should be noted that *E. cloacae* is resistant to cefuroxime, the prophylactic antibiotic used at our institution; this microorganism is able to thrive in humid environments, including water and soil and particularly in healthcare settings. Future studies should investigate care bundle interventions that could prevent Gram-negative SSI-CRANs.

We did not find any relationship between age, gender, emergency procedures, antibiotic prophylaxis, surgical site and duration of surgery. Likewise, and in agreement with other studies [4, 26–29], the NNIS index was not associated with the SSI-CRAN occurrence. [30]. This score is useful in predicting the acquisition of surgical site infection in other procedures but not for craniotomies, probably because most procedures are clean and the duration of surgery does not vary greatly according to reason for surgery. In our study, immunosuppression was not associated with SSI-CRAN. Previous studies have reported conflicting results: some researchers found an association between prolonged use of steroids or chemotherapy with the risk of surgical site infection [6, 31], but others did not [32].

We found that the only independent risk factors for SSI-CRAN were ASA score > 2 and re-intervention. Both variables are well known risk factors for other sites of surgical infection [32–36] and were also identified in previous studies analysing risk factors for SSI-CRAN [1, 4]. The ASA score includes patients’ baseline status, taking into account their comorbidities. In agreement with previous studies [1, 4, 33–37], the SSI-CRAN rates were significantly higher in patients with ASA > 2. Like other researchers [1, 38, 39], we found that patients with re-intervention had an increased risk for infection; therefore, more attention should be paid to patients with the above mentioned risk factors, hardly modifiable by neurosurgeons in most cases.

The strengths of this study are the large number of patients included and the prospective data collection procedure using an internationally accepted definition of surgical site infection and a well-defined follow-up period according to the guidelines [14], which allows reliable comparisons of our results. The fact that a well-trained infection control team oversaw the surveillance programme was another strength. However, there are certain limitations that should be acknowledged. For example, the study was conducted at a single centre, and our findings need to be validated by other studies. Nevertheless, our neurosurgery department is a reference centre in Catalonia and serves a population of over 1,500,000 inhabitants.

## Conclusion

The risk factors and causative agents of SSI-CRAN identified here should be considered when designing preventive strategies aimed to reduce the incidence of this serious complication.

## Additional files

Additional file 1: STROBE Statement—Checklist of items that should be included in reports of cohort studies. (DOCX 18 kb)

Additional file 2: Rewiew of large studies assessing SSI-CRAN. (DOCX 18 kb)

## Abbreviations

ASA: American Society of Anesthesiologists
CDC: Centers for Disease Control and Prevention
CLSI: Clinical Laboratory Standard Institute
CNS: Coagulase Negative Staphylococci
CSF: CerebroSpinal Fluid
GNB: Gram-negative bacilli
GPC: Gram-positive cocci
ICP: IntraCranial Pressure
MRSA: Methicillin-resistant *Staphylococcus aureus*
MSSA: Methicillin-susceptible *Staphylococcus aureus*
NNIS: National Nosocomial Infections Surveillance
SSI-CRAN: Surgical Site Infection after Craniotomy

## References

[1] Fang C, Zhu T, Zhang P, Xia L, Sun C (2017). Risk factors of neurosurgical site infection after craniotomy: a systematic review and meta-analysis. Am J Infect Control.

[2] Davies BM, Jones A, Patel HC (2016). Implementation of a care bundle and evaluation of risk factors for surgical site infection in cranial neurosurgery. Clin Neurol Neurosurg.

[3] Abu Hamdeh S, Lytsy B, Ronne-Engström E (2014). Surgical site infections in standard neurosurgery procedures– a study of incidence, impact and potential risk factors. Br J Neurosurg.

[4] Chiang H-Y, Kamath AS, Pottinger JM, Greenlee JDW, Howard MA, Cavanaugh JE (2014). Risk factors and outcomes associated with surgical site infections after craniotomy or craniectomy. J Neurosurg.

[5] Abode-Iyamah KO, Chiang H-Y, Winslow N, Park B, Zanaty M, Dlouhy BJ (2018). Risk factors for surgical site infections and assessment of vancomycin powder as a preventive measure in patients undergoing first-time cranioplasty. J Neurosurg.

[6] Schipmann S, Akalin E, Doods J, Ewelt C, Stummer W, Suero Molina E (2016). When the infection hits the wound: matched case-control study in a neurosurgical patient collective including systematic literature review and risk factors analysis. World Neurosurg.

[7] O’Keeffe AB, Lawrence T, Bojanic S (2012). Oxford craniotomy infections database: a cost analysis of craniotomy infection. Br J Neurosurg.

[8] Shi Z-H, Xu M, Wang Y-Z, Luo X-Y, Chen G-Q, Wang X (2017). Post-craniotomy intracranial infection in patients with brain tumors: a retrospective analysis of 5723 consecutive patients. Br J Neurosurg.

[9] Kimchi G, Stlylianou P, Wohl A, Hadani M, Cohen ZR, Zauberman J (2016). Predicting and reducing cranioplasty infections by clinical, radiographic and operative parameters – a historical cohort study. J Clin Neurosci.

[10] Sneh-Arbib O, Shiferstein A, Dagan N, Fein S, Telem L, Muchtar E (2013). Surgical site infections following craniotomy focusing on possible post-operative acquisition of infection: prospective cohort study. Eur J Clin Microbiol Infect Dis.

[11] Cheng Y-K, Weng H-H, Yang J-T, Lee M-H, Wang T-C, Chang C-N (2008). Factors affecting graft infection after cranioplasty. J Clin Neurosci.

[12] Erman T, Demirhindi H, Göçer Aİ, Tuna M, İldan F, Boyar B (2005). Risk factors for surgical site infections in neurosurgery patients with antibiotic prophylaxis. Surg Neurol.

[13] Campbell JP, Plaat F, Checketts MR, Bogod D, Tighe S, Moriarty A (2014). Safety guideline: skin antisepsis for central neuraxial blockade. Anaesthesia.

[14] CDC. 9th Surgical Site Infection (SSI) Event 2018. https://www.cdc.gov/nhsn/pdfs/pscmanual/9pscssicurrent.pdf. Accessed 29 Oct 2018.

[15] Charlson ME, Pompei P, Ales KL, MacKenzie CR (1987). A new method of classifying prognostic comorbidity in longitudinal studies: development and validation. J Chronic Dis.

[16] Patel JB, Cockerill FR, Bradford PA, Eliopoulos GM, Hindler JA, Jenkins SG (2012). Performance standards for antimicrobial susceptibility testing; twenty-fifth informational supplement (M100-S22). Wayne, PA. USA.

[17] López Pereira P, Díaz-Agero Pérez C, López Fresneña N, Las Heras Mosteiro J, Palancar Cabrera A, Rincón Carlavilla ÁL (2017). Epidemiology of surgical site infection in a neurosurgery department. Br J Neurosurg.

[18] Mracek J, Hommerova J, Mork J, Richtr P, Priban V (2015). Complications of cranioplasty using a bone flap sterilised by autoclaving following decompressive craniectomy. Acta Neurochir.

[19] Mollman HD, Haines SJ (1986). Risk factors for postoperative neurosurgical wound infection. J Neurosurg.

[20] Patir R, Mahapatra AK, Banerji AK (1992). Risk factors in postoperative neurosurgical infection. Acta Neurochir.

[21] Hardy SJ, Nowacki AS, Bertin M, Weil RJ (2010). Absence of an association between glucose levels and surgical site infections in patients undergoing craniotomies for brain tumors. J Neurosurg.

[22] Wei Z, Gordon CR, Bergey GK, Sacks JM, Anderson WS (2016). Implant site infection and bone flap osteomyelitis associated with the NeuroPace responsive Neurostimulation system. World Neurosurg.

[23] Meng Y, Voisin MR, Suppiah S, Merali Z, Moghaddamjou A, Alotaibi NM (2018). Risk factors for surgical site infection after intracranial electroencephalography monitoring for epilepsy in the pediatric population. J Neurosurg Pediatr.

[24] Phung J, Mathern GW, Krogstad P (2013). Timing and predictors of fever and infection after craniotomy for epilepsy in children. Pediatr Infect Dis J.

[25] McKerr C, Coetzee N, Edeghere O, Suleman S, Verlander NQ, Banavathi K (2017). Association between post-craniotomy Propionibacterium acnes infection and dural implants: a case–control study. J Hosp Infect.

[26] Chiang H, Steelman VM, Pottinger JM, Schlueter AJ, Diekema DJ, Greenlee JDW (2011). Clinical significance of positive cranial bone flap cultures and associated risk of surgical site infection after craniotomies or craniectomies. J Neurosurg.

[27] Korinek A-M, Golmard J-L, Elcheick A, Bismuth R, van Effenterre R, Coriat P (2005). Risk factors for neurosurgical site infections after craniotomy: a critical reappraisal of antibiotic prophylaxis on 4578 patients. Br J Neurosurg.

[28] Sánchez-Arenas R, Rivera-García BE, Grijalva-Otero I, Juárez-Cedillo T (2010). del Carmen Martínez-García M, Rangel-Frausto S. Factores asociados a infecciones nosocomiales en sitio quirúrgico para craneotomía. Cir Cir.

[29] Lietard C, Thébaud V, Besson G, Lejeune B (2008). Risk factors for neurosurgical site infections: an 18-month prospective survey. J Neurosurg.

[30] Gaynes RP, Culver DH, Horan TC, Edwards JR, Richards C, Tolson JS (2001). Surgical site infection (SSI) rates in the United States, 1992–1998: the National Nosocomial Infections Surveillance System Basic SSI risk index. Clin Infect Dis.

[31] McCutcheon BA, Ubl DS, Babu M, Maloney P, Murphy M, Kerezoudis P (2016). Predictors of surgical site infection following craniotomy for intracranial neoplasms: an analysis of prospectively collected data in the American College of Surgeons National Surgical Quality Improvement Program Database. World Neurosurg.

[32] Malone DL, Genuit T, Tracy JK, Gannon C, Napolitano LM (2002). Surgical site infections: reanalysis of risk factors. J Surg Res.

[33] Cheng K, Li J, Kong Q, Wang C, Ye N, Xia G (2015). Risk factors for surgical site infection in a teaching hospital: a prospective study of 1,138 patients. Patient Prefer Adherence.

[34] Khan M, Rooh-ul-Muqim ZM, Khalil J, Salman M (2010). Influence of ASA score and Charlson comorbidity index on the surgical site infection rates. J Coll Physicians Surg Pak.

[35] Wloch C, Wilson J, Lamagni T, Harrington P, Charlett A, Sheridan E (2012). Risk factors for surgical site infection following caesarean section in England: results from a multicentre cohort study. BJOG An Int J Obstet Gynaecol.

[36] Stanic S, Bojanic J, Grubor P, Mijovic B, Maric V (2017). Examination of risk factors for the development of surgical site infections. Mater Sociomed.

[37] Merkler AE, Saini V, Kamel H, Stieg PE (2014). Preoperative steroid use and the risk of infectious complications after neurosurgery. The Neurohospitalist.

[38] Chen C, Zhang B, Yu S, Sun F, Ruan Q, Zhang W (2014). The incidence and risk factors of meningitis after major craniotomy in China: a retrospective cohort study. PLoS One.

[39] Korinek A-M, Baugnon T, Golmard J-L, van Effenterre R, Coriat P, Puybasset L (2008). Risk factors for adult nosocomial meningitis after craniotomy. Neurosurgery.

