# Supplementary material for: Risk factors for surgical site infection after craniotomy: a prospective cohort study
Source: Antimicrob Resist Infect Control. 2019 May 2;8:69. doi: 10.1186/s13756-019-0525-3 (PMC6498621; doi:10.1186/s13756-019-0525-3)
Supplement: Supplementary file 2 — Rewiew of large studies assessing SSI-CRAN. (DOCX 18 kb) [file 13756_2019_525_MOESM2_ESM.docx]

Supplementary Material: *Rewiew of large studies assessing SSI-CRAN*

| Study | Year | Origin Country | Study Period | Infection/Case patients (%) | Independent Risk Factors for SSI-CRAN |
| --- | --- | --- | --- | --- | --- |
| Korinek et al^1^ | 2005 | France | May 1997 - December 2001 | 303 /4578 (6.6) | - CSF leak - Male gender - Surgical diagnosis - Surgeon - Early re-operation - Surgical duration - Absence of prophylaxis |
| Lietard et al^2^ | 2008 | France | September 2000 - March 2002 | 35/884 (3.9) | - CSF leak - External shunt - Altemeier class - Further neurosurgery |
| Sánchez-Arenas et al^3^ | 2010 | Mexico | January 2000 – December 2001 | 41/737 (5.6) | - Chronic diseases - Non-traumatic causes of surgery - Late shift procedures |
| Sneh-Arbib et al^4^ | 2013 | Israel | October 2010 and April 2012 | 28/502 (5.6) | - Non-elective surgery - External CSF drainage - Re-operation - Post-operative respiratory failure |
| Abu Hamdeh et al^5^ | 2014 | Sweden | January – December 2010 | 23/448 (5.1) | - Meningioma - Longer operation time |
| Chiang et al^6^ | 2014 | USA | January 2006 – December 2010 | 104/2541 (4.1) | - Preoperative SSI risk index - Previous brain operation - Body mass index - Chemotherapy on admission - Preoperative hospital stay - Procedure reason - Preoperative glucose level |
| Davies et al^7^ | 2016 | UK | October 2011 – February 2013 | 66/1253 (5.3) | - Implantation of foreign material |
| Schipmann et al^8^ | 2016 | Germany | January 2012 - March 2015 | 70/2819 (2.5) | - ASA score - Surgical drain - Previous operations - Implantation of foreign material |
| Abode-Iyamah et al^9^ | 2017 | USA | January 2008 - July 2014 | 15/258 (5.8) | - Diabetes - Multiple craniotomy procedures - Prior same-side craniotomy - Implantation of foreign material |
| Shi et al^10^ | 2017 | China | January 2012 - December 2013 | 391/5723 (6.8) | - Clean-contaminated craniotomy - Surgical duration - External CSF drainage/leak - Age |

1. Korinek A-M, Golmard J-L, Elcheick A, Bismuth R, van Effenterre R, Coriat P, et al. Risk factors for neurosurgical site infections after craniotomy: a critical reappraisal of antibiotic prophylaxis on 4578 patients. Br J Neurosurg 2005;19:155–62. doi:10.1080/02688690500145639.
2. Lietard C, Thébaud V, Besson G, Lejeune B. Risk factors for neurosurgical site infections: an 18-month prospective survey. J Neurosurg 2008;109:729–34. doi:10.3171/JNS/2008/109/10/0729.
3. Sánchez-Arenas R, Rivera-García BE, Grijalva-Otero I, Juárez-Cedillo T, del Carmen Martínez-García M, Rangel-Frausto S. Factores asociados a infecciones nosocomiales en sitio quirúrgico para craneotomía. Cir Cir 2010;78:5–13.
4. Sneh-Arbib O, Shiferstein A, Dagan N, Fein S, Telem L, Muchtar E, et al. Surgical site infections following craniotomy focusing on possible post-operative acquisition of infection: prospective cohort study. Eur J Clin Microbiol Infect Dis 2013;32:1511–6. doi:10.1007/s10096-013-1904-y.
5. Abu Hamdeh S, Lytsy B, Ronne-Engström E. Surgical site infections in standard neurosurgery procedures– a study of incidence, impact and potential risk factors. Br J Neurosurg 2014;28:270–5. doi:10.3109/02688697.2013.835376.
6. Chiang H-Y, Kamath AS, Pottinger JM, Greenlee JDW, Howard MA, Cavanaugh JE, et al. Risk factors and outcomes associated with surgical site infections after craniotomy or craniectomy. J Neurosurg 2014;120:509–21. doi:10.3171/2013.9.JNS13843.
7. Davies BM, Jones A, Patel HC. Implementation of a care bundle and evaluation of risk factors for surgical site infection in cranial neurosurgery. Clin Neurol Neurosurg 2016;144:121–5. doi:10.1016/j.clineuro.2016.03.025.
8. Schipmann S, Akalin E, Doods J, Ewelt C, Stummer W, Suero Molina E. When the Infection Hits the Wound: Matched Case-Control Study in a Neurosurgical Patient Collective Including Systematic Literature Review and Risk Factors Analysis. World Neurosurg 2016;95:178–89. doi:10.1016/j.wneu.2016.07.093.
9. Abode-Iyamah KO, Chiang H-Y, Winslow N, Park B, Zanaty M, Dlouhy BJ, et al. Risk factors for surgical site infections and assessment of vancomycin powder as a preventive measure in patients undergoing first-time cranioplasty. J Neurosurg 2018;128:1241–9. doi:10.3171/2016.12.JNS161967.
10. Shi Z-H, Xu M, Wang Y-Z, Luo X-Y, Chen G-Q, Wang X, et al. Post-craniotomy intracranial infection in patients with brain tumors: a retrospective analysis of 5723 consecutive patients. Br J Neurosurg 2017;31:5–9. doi:10.1080/02688697.2016.1253827.
